# Expression of Apoptosis-Related Genes in Cat Testicular Tissue in Relation to Sperm Morphology and Seasonality—A Preliminary Study

**DOI:** 10.3390/ani11020489

**Published:** 2021-02-12

**Authors:** Sylwia Prochowska, Agnieszka Partyka, Wojciech Niżański

**Affiliations:** Department of Reproduction and Clinic of Farm Animals, Wroclaw University of Environmental and Life Sciences, 50-366 Wroclaw, Poland; agnieszka.partyka@upwr.edu.pl (A.P.); wojciech.nizanski@upwr.edu.pl (W.N.)

**Keywords:** feline semen, teratozoospermia, proapoptotic genes, antiapoptotic genes, caspases

## Abstract

**Simple Summary:**

Programmed cell death (apoptosis) is a crucial process in spermatogenesis, responsible for the elimination of abnormal sperm cells and for the reduction of testicular volume outside the breeding season. Poor sperm morphology (teratozoospermia) and lower semen quality out of season are commonly observed in domestic cats, but the exact reasons and mechanisms are not known. The aim of this study was to use gene expression analysis to identify which apoptotic processes and pathways are expressed in the phenomenon of teratozoospermia and seasonality in the domestic cat. The results showed a higher expression of two antiapoptotic genes and one proapoptotic gene during the non-reproductive season, with no differences noted between normozoospermic and teratozoospermic cats. We hypothesize that during the non-breeding season there is a potential detrimental factor which activates a cascade of caspases, against which germ cells mount a defense by producing anti-apoptotic proteins. Further identification of this factor may help in the amelioration of semen quality of cats and improve feline breeding.

**Abstract:**

Apoptosis is a crucial process in spermatogenesis, responsible for the elimination of abnormal sperm cells and testicular regression out of breeding season. The aim of this study was to assess if the expression of apoptosis-related genes in testicular tissue of domestic cats differed: (1) between normozoospermic and teratozoospermic donors, and (2) between reproductive and non-reproductive season. The expression of genes: *BCL2L1*, *BCL2, BAX, BAD, FAS*, *FASLG*, and caspases (*CASP3, CASP8, CASP9*, and *CASP10*) was analyzed by qRT-PCR in testicular tissue samples. During non-reproductive season significantly higher expression of two anti-apoptotic genes (*BCL2L1* and *BCL2*) was observed. Additionally, there was a significant higher expression of *CASP10* in teratozoospermic cats during non-reproductive than during reproductive season. No differences were noted between normozoospermic and teratozoospermic groups. Upregulation of some genes during the non-reproductive season indicates engagement of apoptotic mechanisms in the seasonal changes of semen quality in cats, however further studies on protein levels and analysis of changes on distinct testicular germinal layers are required. At the same time, teratozoospermia in the general population of cats seems to be not connected with dysregulation of apoptosis in the testes.

## 1. Introduction

Apoptosis, one of the types of programmed cell death, is a crucial process involved in the normal development and maintenance of homeostasis of every organism. Numerous studies have demonstrated its important role in spermatogenesis (reviewed by [1]). Physiologically, in adult males apoptosis is involved in: balancing the ratio of germ cells per Sertoli cell [2]; elimination of defective germ cells that cannot complete meiosis [3]; and removal of cytoplasm during spermatid maturation [4]. In many seasonally-breeding species, programmed cell death is one of the main processes responsible for testicular regression during reproductive quiescence (reviewed by [5]). In addition to physiological conditions, various pathological factors can trigger germ cell apoptosis, e.g., increased temperature [6], toxins [7], chemotherapeutics [8], irradiation [9], and deprivation of gonadotropins [10,11].

Little is known about apoptosis in the testes of domestic cats. It has been studied in relation to age and breeding season [12], revealing significant age-related, but not seasonal, differences. However, seasonality in male cats is a controversial subject, with some authors reporting seasonal changes in testis weight, testosterone concentration, and semen quality [13,14] that were not observed by the others [15,16]. In the Siemieniuch study [12], information about testicular weight and semen quality was not provided. Therefore, apoptotic processes may still be involved in the regulation (or dysregulation) of spermatogenesis out of the reproductive season, when changes in semen quality are noted. This aspect needs further investigation.

Reduced germ cell apoptosis has been proposed as an explanation for the high incidence of teratozoospermia (the presence of >60% morphologically abnormal cells in the ejaculate [17]) in feline species [18]. According to this hypothesis, disturbances in germ cell depletion during spermatogenesis result in the higher presence of malformed sperm cells in the ejaculate (due to persistence of abnormal sperm cells, which should have been eliminated via apoptosis). This can be observed as a higher number of sperm cells per Sertoli cell [18,19]. This type of teratozoospermia was found in highly inbred rare wild felids (e.g., Florida panthers, clouded leopards, and cheetahs) and an experimentally inbred colony of domestic cats [20]. However, there is also another type of teratozoospermia, observed in a random population of young cats. These males showed a high percentage of malformed cells, but with normal germ cell to Sertoli cell ratio [21]. The etiology of this type of teratozoospermia is unknown and the role of apoptosis has not yet been evaluated.

In the studies mentioned above [12,18] apoptosis was measured at the effector stage (DNA fragmentation). This approach provides information about the intensity of apoptosis, but not about the apoptotic pathways engaged, nor their regulation. The use of the qPCR technique may shed some light on how the apoptosis is mediated during spermatogenesis in the domestic cat.

The aim of this study was to assess if the expression of apoptosis-related genes in testicular tissue of domestic cats differ: (1) between normozoospermic and teratozoospermic donors, and (2) between reproductive and non-reproductive season.

## 2. Materials and Methods

### 2.1. Animals and Sample Preparation

The study samples consisted of testicular tissue obtained from 22 privately owned cats after a routine castration at the Department of Reproduction and Clinic of Farm Animals, Wrocław University of Environmental and Life Sciences, Poland. The tomcats were mixed-breed, aged between 8 months and 4 years. The cats were not used for breeding and were healthy at the moment of castration. Samples (100 mg of tissue from each testis) were kept in RNALater^®^ solution at 5 °C for 24 h, then snap frozen and stored at –80 °C until RNA isolation. Depending on the month when the orchidectomy was performed, the samples were divided into two groups: collected during the reproductive season (March–September in the northern hemisphere) and collected during the non-reproductive season (December–February in the northern hemisphere). Additionally, urethral semen was collected by the Zambelli method [22] before castration. Sperm motility was assessed subjectively under a phase contrast microscope (magnification ×200) and sperm morphology was assessed after eosin-nigrosine staining as previously described [16]. The cats were classified as normozoospermic when there were >60% normal spermatozoa in the semen sample, or teratozoospermic when <60% normal spermatozoa were present. All procedures were performed with the agreement of the Second Local Ethical Committee in Wroclaw.

### 2.2. Study Design

**Figure 1:**
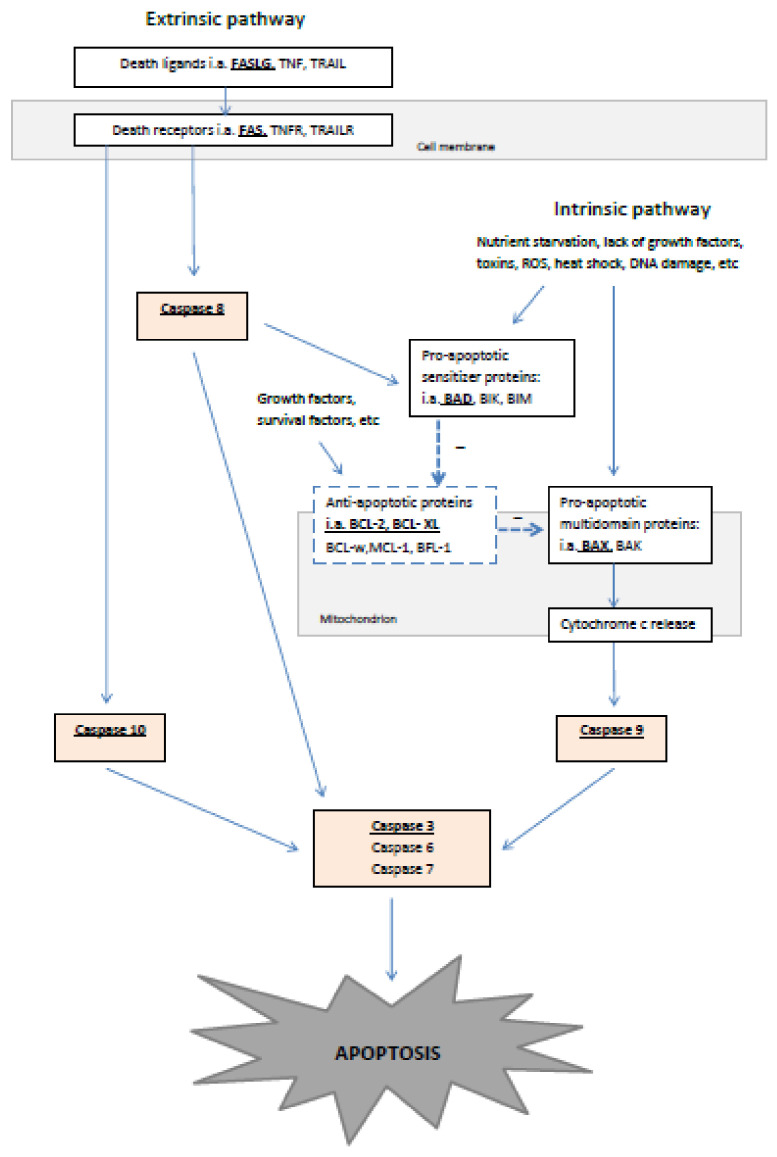
Apoptotic signaling via two main pathways: extrinsic and intrinsic. All the genes examined in this study are highlighted (bold and underlined).

The study was divided into two experiments. All the examined genes and their position in the apoptotic signaling are depicted in Figure 1.

#### 2.2.1. Experiment I—Expression of Genes from BCL-2 Family

In the first experiment, the relative expression of two anti-apoptotic genes (*BCL2L1* and *BCL2*) and two pro-apoptotic genes (*BAX* and *BAD*) was quantified in 12 testicular samples. Since there were not enough cats to perform a 2 × 2 factorial analysis (normozoospermic, reproductive season *n = 4*, normozoospermic, non-reproductive season *n = 1*, teratozoospermic, reproductive season *n = 3*, teratozoospermic, non-reproductive season *n = 4*), these two variables were analyzed separately. Samples were allocated to the normozoospermic (*n = 5*) or teratozoospermic (*n = 7*) group, based on the sperm morphology results. A separate, parallel analysis was conducted for the same genes in the same samples, based on whether the samples were collected during the reproductive season *(n = 5*) or the non-reproductive season (*n = 7*).

#### 2.2.2. Experiment II—Expression of Genes of FAS/FAS-Ligand and Caspases

In the second experiment, we focused on the expression of genes connected with death receptor signaling: Fas cell surface death receptor (*FAS*) and Fas ligand (*FASLG*); and with the caspase cascade: caspase 3 (*CASP3*), caspase 8 (*CASP8*), caspase 9 (*CASP9*), and caspase 10 (*CASP10*). In this experiment, sperm morphology and seasonality were analyzed simultaneously. Eighteen samples were divided into three groups: normozoospermic in the reproductive season (*n = 6*), teratozoospermic in the reproductive season (*n = 6*), and teratozoospermic in the non-reproductive season (*n = 6*). During the non-reproductive season, none of the cats were classified as normozoospermic.

### 2.3. Gene Expression Analysis

#### 2.3.1. Isolation of RNA and Reverse Transcription

Testicular tissue (100 mg) was homogenized in TRIzol^®^ (TRIzol^®^ Plus RNA Purification System, Invitrogen) using TissueRuptor (Qiagen GmbH, Hilden, Germany). Total RNA was extracted using the TRIzol^®^ method in combination with a column-based RNA isolation method (PureLink^TM^ RNA Mini Kit, Invitrogen). DNase treatment (PureLink^®^ DNase Set, Invitrogen) was performed to eliminate contaminating genomic DNA from the samples. To test the RNA quantity and quality, the isolated total RNA was checked by spectrophotometric method (DeNovix Inc., Wilmington, DE, USA). Subsequently, single-strand cDNA synthesis was conducted using the iScript™ cDNA Synthesis Kit (BioRad laboratories Inc., Hercules, CA) with blend of oligo(dT) and random hexamer primers and a modified Moloney murine leukemia virus (MMLV) reverse transcriptase, according to the manufacturer’s instructions. The generated cDNA served as the template for qRT PCR and was stored at –80 °C until use.

Negative controls omitting the reverse transcriptase enzyme were performed to exclude any genomic DNA contamination in the extracted RNA.

#### 2.3.2. RT-qPCR Technique

Primers that were used in this study are shown in Table 1.

Primer pairs for the studied genes were designed by Primer-BLAST (http://blast.ncbi.nlm.nih.gov/Blast.cgi) using sequences published in GenBank (http://www.ncbi.nlm.nih.gov/), which are available online.

The expression of the target genes was quantified using the comparative threshold cycle (Ct) method where the expression was normalized to an internal control (in Exp. I: 40 S ribosomal protein S7–*RPS7* and in Exp. II: glyceraldehyde-3-phosphate dehydrogenase–*GAPDH*) and expressed relative to a calibrator sample (a sample collected during the reproductive season from a tomcat with the highest proportion of normal spermatozoa).

Gene expression was measured by Real-Time quantitative PCR (RT-qPCR) using the CFX96 System (BioRad Laboratories Inc., Hercules, CA) and amplified using SsoAdvanced™ Universal SYBR^®^ Green Supermix (BioRad Laboratories Inc., Hercules, CA). RT-qPCR was performed under the following conditions: 95 °C for 30s, followed by 40 cycles of 95 °C for 15s, and 60 °C for 1 min. Following each PCR, melting curves were generated by stepwise increases in temperature from 65 to 95 °C to ensure single product amplification. All assays for each gene were run in duplicate in the same reaction using 50 ng cDNA sample together with the no template control. The obtained melting points of the amplicons served as confirmation for specific amplifications. A single peak at the melting temperature of the PCR-product confirmed primer specificity. Reactions containing no template (NTC) (H_2_O) were used to verify that obtained amplicons in the real-time PCR were not derived from contaminations. Expression of each target gene was normalized to the *RPS7* (Exp. I) or *GAPDH* (Exp. II) and relative differences in gene expression were calculated using the Pfaffl method. Amplification efficiency (E) was calculated from the plot of the Cq values against cDNA input according to the equation E = 10^(−1/slope)^ [23]. The amplification efficiency (%E) of each gene was: *RPS7*–100.4%, *GAPDH*–96.4%, *BCL2L1*–106.9%, *BCL2*–110.8%, *BAD*–01.7%, *BAX*–103.1%, *FAS*–101.6%, *FASLG*–97.4%, *CASP3*–99.2%, *CASP8*–not found, *CASP9*–95.3%, *CASP10*–100.1%. The relative expression ratio of a target gene in comparison with a reference gene was calculated according to the equation R = E_target_^ΔCq target (calibrator–sample)^/E_reference_^ΔCq reference (calibrator–sample)^ [24].

### 2.4. Data Analysis

Data distribution was analyzed by the Shapiro-Wilk test and variance homogeneity was analyzed by the Brown-Forsythe test. Statistical analysis was performed using the Student t-test (Experiment I) or by ANOVA and Duncan Test (Experiment II). The significance level was set at *p* < 0.05.

## 3. Results

The quality of semen of the cats in the study is shown in Table 2 (Experiment I) and Table 3 (Experiment II).

There was a clear deterioration in semen quality out of the reproductive season–only one cat in Exp. I (data not shown) and none of the cats in Exp. II were classified as normozoospermic in the period from December to February.

All examined genes were expressed in feline testicular tissue, except caspase 8. A laboratory error can be excluded, as the same primers were used in another experiment and expression of caspase 8 was found in examined tissues (uterus, skeletal muscle, and lymph node, data not shown).

**Figure 2:**
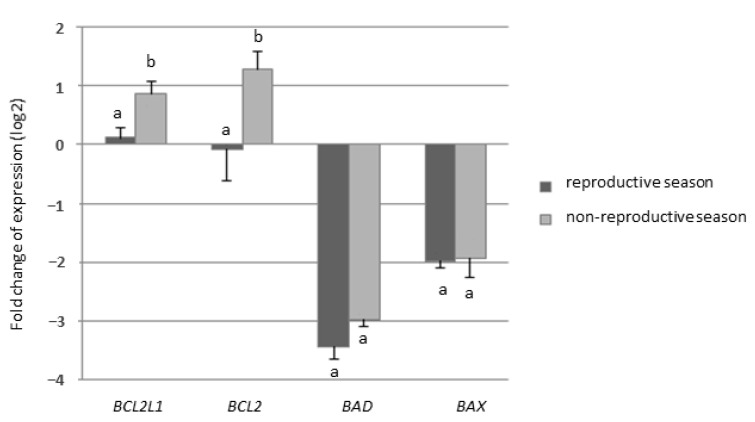
Results of Experiment I. Relative gene expression of antiapoptotic (*BCL2L1, BCL2*) and proapoptotic (*BAX, BAD*) genes in testicular tissue collected during the reproductive and non-reproductive season. a, b: different lowercase letters indicate significant differences (*p* < 0.05). Data presented as mean values ± SEM.

In Experiment I, no differences were found between normo- and teratozoospermic groups for any of genes analyzed (*p* > 0.05). Comparison of samples collected during the reproductive and non-reproductive season revealed significantly higher expression of two anti-apoptotic genes (*BCL2L1* and *BCL2*) during the non-reproductive season (*p* < 0.05) (Figure 2). For pro-apoptotic genes (*BAX* and *BAD*) no significant differences between reproductive and non-reproductive season were found (*p* > 0.05) (Figure 2).

**Figure 3:**
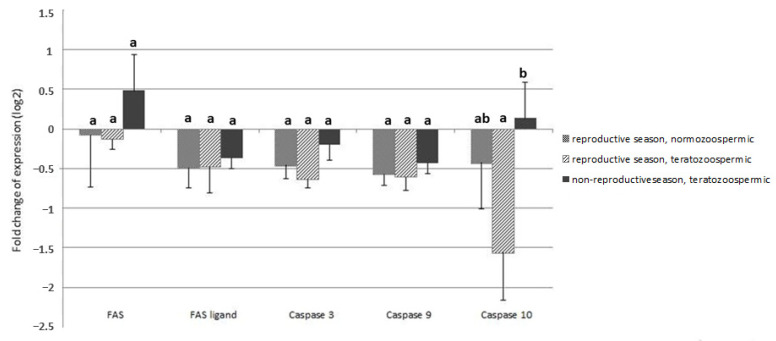
Results of Experiment II. Relative expression of genes involved in apoptotic cascade: *FAS*, *FASLG* and chosen caspases in testicular tissue of normozoospermic and teratozoospermic donors during the reproductive and non-reproductive season. a, b: different lowercase letters indicate significant differences (*p* < 0.05). Data presented as mean values ± SEM.

In Experiment II, significantly higher expression of *CASP10* was found in the teratozoospermic cats during the non-reproductive season compared to the reproductive season (Figure 3, *p* < 0.05). The expression of other genes was not different between the three study groups (*p* > 0.05).

## 4. Discussion

In this study we used gene expression analysis to identify which (if any) apoptotic processes and pathways are involved in the phenomenon of teratozoospermia and seasonal changes of semen quality in the domestic cat. The results showed that in the general cat population, teratozoospermia is not connected with dysregulation of apoptosis in the testes at the gene expression level. On the other hand, differential expression of some pro- and antiapoptotic genes during the non-reproductive season may indicate engagement of apoptotic mechanisms in the seasonal deterioration of semen quality in cats, which needs further investigation.

Teratozoospermia in felids is a well-known, commonly observed phenomenon that was confirmed in this study, as even males in the normozoospermic group had sperm morphology values that were just above the threshold of 60%. There are two types of feline teratozoospermia [21]; one type is seen in highly inbred populations, while the other is observed in random populations of cats. Our study group consisted of cats (both teratozoospermic and normozoospermic) whose inbreeding level was not known. Due to factors outside the authors’ control, the analysis of Sertoli cell efficiency, which helps in distinguishing between these two types of teratozoospermia [21], was not possible. However, as the material was collected from a random population, we assumed that our teratozoospermic samples represented the second type of teratozoospermia, which has an unknown etiology. Studies comparing normo- and teratozoospermic cats have suggested some potential factors, such as hormonal imbalance in the testes [24], diet and stress [25], age and season [26]. Some of these factors (e.g., hormone depletion, stress, toxicants) may induce testicular apoptosis [27]. Depending on whether the damaging factor works on the germ cells themselves or via the Sertoli cells, different expression of genes involved in the intrinsic (Bcl-2-family proteins, caspase 9) and extrinsic (Fas, Fas ligand, and caspase 8 and 10) pathway can be observed [28] (Figure 1). In our study, there were no differences in the expression level of any of these genes between normozoospermic and teratozoospermic cats. This could suggest that the second type of teratozoospermia is not caused by a damaging factor or that this factor is not working via apoptosis. It would be interesting to analyze gene expression in the case of teratozoospermia caused by various agents under controlled conditions.

The second aspect analyzed—seasonality in male cats—is a controversial subject and the results of different studies are contradictory [13,15,29]. In our previous study [16], we did not find significant changes in the quality of semen collected during the breeding and non-breeding seasons. However, in this study the percentage of morphologically normal sperm cells was markedly lower during the non-reproductive season and none of the cats in the Experiment II were classified as normozoospermic in December–February period. These findings seem to confirm the occurrence of seasonal variations in semen quality in cats.

A higher expression of caspase 10 in the teratozoospermic cats during the non-breeding season could suggest an activation of germ cell apoptosis generated by an extrinsic pathway. At the same time, during non-reproductive season we observed a higher expression of anti-apoptotic genes *BCL2L1* and *BCL2*, which are connected with an intrinsic apoptotic pathway. Upregulation of these genes results in decreased apoptosis. The lack of difference for caspase 9 (mediating an intrinsic apoptotic pathway, connected to BCL-2 family proteins, Figure 1), and for effector caspase 3 in teratozoospermic cats during the non-reproductive season could suggest that the activation of antiapoptotic proteins successfully counter-balances the detrimental effect of the unknown factor inducing apoptosis via the extrinsic pathway. Our results are therefore in agreement with the study of Siemieniuch [12], which did not report any changes in the level of apoptosis based on the reproductive season, using DNA fragmentation (an endpoint of apoptosis, evaluated by TUNEL assay) as a marker of programmed cell death. Therefore, it could be that here we have observed a compensatory defense mechanism, similar to that proposed for type one teratozoospermia because some germ cells die via apoptosis mediated by caspase 10, while in other cells anti-apoptotic mechanisms are activated to increase the sperm output at the expense of sperm quality. This hypothesis may explain the lower semen quality during the non-breeding season reported here and by other authors. However, although the selection of genes in this study allowed us to distinguish processes connected to the extrinsic and intrinsic pathways, it could not indicate precisely which cells were affected. More comprehensive immunohistochemical studies are now required to evaluate which spermatogenic cells (e.g., spermatogonia, spermatids) are involved.

Surprisingly, we did not find an expression of caspase 8 in the feline testicular tissue. In humans it has been demonstrated that this caspase has the lowest expression in testis, muscles and brain [30]. Possibly the same situation occurs in cats, but further studies in this subject are required.

## 5. Conclusions

Based on our results, we can conclude that different apoptotic pathways are activated in the feline testis during the non-reproductive season. We suggest that there is a potential detrimental factor, against which germ cells mount a defense by producing anti-apoptotic proteins. Identification of this factor and its target cells in the testis may be the next step in the studies about male cat reproductive seasonality.

On the other hand, it seems that contrary to highly inbred cats, apoptosis is not involved in origination of teratozoospermia in a random population of cats. Therefore, the mechanism underlying the phenomenon of poor semen quality in cats remains unresolved and aspects other than apoptosis should be further evaluated.

## Figures and Tables

**Table 1:** Primer sequence for quantitative real-time polymerase chain reaction amplification of mRNA from feline testes.

Name	Accession No.	Primers Sequence (5′–3′)	Amplicon Size (bp)
*RPS7*	NM_001009832.1	F: GGGCAAGAGAATCCGTGTGA R: CCTTGCCCGTGAGCTTCTTA	131
*GAPDH*	NM_001009307.1	F: GGAGAAAGCTGCCAAATATG R: CAGGAAATGAGCTTGACAAAGTGG	192
*BCL2L1*	NM_001009228.1	F: GCTTGGATGGCCACTTACCT R: TGCTGCATTGTTCCCGTAGA	99
*BCL2*	NM_001009340.1	F: GGAGGATTGTGGCCTTCT R: GTTATCCTGGATCCAGGTGT	143
*BAX*	NM_001009282.2	F: GCTCTGAGCAGATCATGAAGACA R: CATTCGCCCTGCTCGATCTT	71
*BAD*	XM_003993558.3	F: GGGCTCCTTCAAGGGACTTC R: TCCTCTCCCCAAGTTCCGAT	118
*FAS*	NM_001009314.1	F: GCTCCTGATTCTACCGTCCG R: CCGGAGCAGTTGGACTTTCT	283
*FASLG*	NM_001009352.1	F: TCCACCAGCCAAAAGCATGT R: TTGAGTTGGGCTTGCCTGTT	118
*CASP3*	NM_001009338.1	F: CCGGCAAACCCAAACTCTTC R: AACCAGGGGCTGTGGAATAC	153
*CASP8*	XM_006935474.2	F: CGCTTCTTTGGTAAGGCTACA R: GGATGTAGTCCAGGCTCAGG	140
*CASP9*	XM_011284592.1	F: CTAGTTTGCCCACACCCAGT R: ACAGCATTAGCGACCCTGAG	175
*CASP10*	XM_006935472.2	F: CCGAGCATTCACCTCCTACC R: TCAGTCCGGGGAAAACCAAC	435

**Table 2:** Semen parameters of cats in Experiment I. Data presented as mean ± SD.

Study Group	According to Morphology		According to Season	
	Normospermic (*n = 5*)	Teratospermic (*n = 7*)	Reproductive (*n = 5*)	Non-Reproductive (*n = 7*)
Subjective motility [%]	80.0 ± 7.1	65.7 ± 15.1	74.3 ± 11.3	68.0 ± 17.9
MORPHOLOGY				
Normal [%]	66.2 ± 5.8	31.1 ± 13.5	50.9 ± 20.4	38.6 ± 21.7
Distal droplet [%]	11.6 ± 7.7	11.2 ± 9.3	14.1 ± 8.0	7.6 ± 7.9
Bent tail [%]	5.7 ± 5.8	18.3 ± 19.4	8.2 ± 9.4	19.8 ± 22.0
Detached head [%]	1.8 ± 0.7	1.6 ± 1.3	1.6 ± 0.9	1.7 ± 1.3
Coiled tail [%]	0.1 ± 0.2	0.3 ± 0.5	0.1 ± 0.2	0.4 ± 0.5
Proximal droplet [%]	3.0 ± 2.2	4.8 ± 7.5	5.6 ± 7.2	1.9 ± 1.5
Head abnormalities [%]	1.8 ± 0.4	3.5 ± 3.0	2.0 ± 1.9	3.9 ± 2.8
Acrosome abnormalities [%]	6.1 ± 6.1	7.1 ± 3.7	6.1 ± 5.3	7.4 ± 4.0
Midpiece defects [%]	3.4 ± 3.1	19.5 ± 8.1	10.2 ± 9.5	16.4 ± 11.6
Dag-like defect [%]	0.3 ± 0.7	2.6 ± 1.8	1.2 ± 1.7	2.3 ± 2.1

**Table 3:** Semen parameters of cats in Experiment II. Data presented as mean ± SD.

Study Group:	Normospermic in Reproductive Season (*n = 6*)	Teratospermic in Reproductive Season (*n = 6*)	Teratospermic in Non- Reproductive Season (*n = 6*)
Subjective motility [%]	81.7 ± 7.5	77.5 ± 11.7	53.3±19.7
MORPHOLOGY Normal [%]	65.6 ± 5.6	38.6 ± 9.7	21.5 ± 7.6
Distal droplet [%]	8.8 ± 6.6	14.5 ± 9.2	11.8 ± 14.3
Bent tail [%]	4.8 ± 5.6	6.3 ± 4.2	20.3 ± 18.9
Detached head [%]	2.0 ± 1.0	2.1 ± 3.0	4.0 ± 7.5
Coiled tail [%]	0.1 ± 0.2	0.3 ± 0.6	0.9 ± 0.9
Proximal droplet [%]	2.8 ± 2.0	11.0 ± 8.0	4.4 ± 5.7
Head abnormalities [%]	1.5 ± 0.5	4.6 ± 4.6	5.8 ± 3.8
Acrosome abnormalities [%]	6.2 ± 5.6	6.4 ± 3.0	6.1 ± 3.9
Midpiece defects [%]	6.6 ± 5.6	13.8 ± 10.5	19.8 ± 8.4
Dag-like defect [%]	1.7 ± 3.2	2.5 ± 1.4	5.2 ± 4.8

## Data Availability

The data that support the findings of this study are available from the corresponding author (S.P.), upon reasonable request.
